# A Mechanism of Food‐Web Complexity Emergence Under Multiple Environmental Drivers

**DOI:** 10.1002/ece3.73138

**Published:** 2026-02-20

**Authors:** Guanming Guo, Helin Zhang

**Affiliations:** ^1^ Key Laboratory of Low Altitude Geographic Information and Air Route of Jiangxi Education Institutes Jiangxi Flight University Nanchang Jiangxi China; ^2^ Ministry of Education Key Laboratory for Transboundary Ecosecurity of Southwest China Yunnan University Kunming China; ^3^ Key Laboratory of Poyang Lake Wetland and Watershed Research, School of Geography and Environment Jiangxi Normal University Nanchang Jiangxi China

**Keywords:** colonization‐competition tradeoff, disturbance, ecosystem size, food web complexity, multiple environmental factors, resource productivity

## Abstract

Understanding the mechanisms that drive the emergence and maintenance of food‐web complexity under multiple environmental drivers is a central challenge in metacommunity ecology. The environmental variables (ecosystem size, resource productivity, and disturbance) are often considered as potential drivers of complex ecosystems. However, how these factors shape food‐web complexity remains unclear. Here, we develop a colonization‐competition tradeoff framework for complex ecological network metacommunities including multiple environmental drivers. We found that the topological complexity of ecological networks, measured by species richness, connectance, omnivory, and mean food chain length, exhibits robust, non‐linear idiosyncratic responses to gradients in ecosystem size, resource productivity, or disturbance. The non‐monotonicity arises from shifts in the dominance hierarchy of basal species driven by colonization‐competition tradeoffs, which in turn cascade upwards to alter consumer community composition and network structure. Moreover, this result stays valid even when parameters are altered or the assumption of a strict competitive hierarchy is relaxed. It reveals that a minor change in these environmental determinants can result in significant implications for the complexity of trophic networks. Thus, this study offers a mechanistic explanation for the emergence of food‐web complexity driven by multiple environmental drivers.

## Graphical abstract

This study demonstrates that the topological complexity of ecological networks, including metrics like species richness and connectance, exhibits nonlinear oscillatory responses to changes in ecosystem size, productivity, and disturbance. These findings reveal that even minor environmental alterations can significantly impact food‐web complexity, providing a mechanistic explanation for its emergence under multiple environmental drivers.

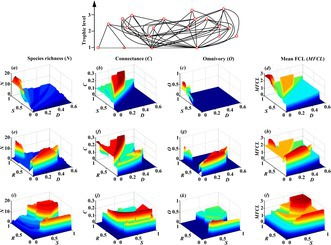

## 1 Introduction

How food web complexity arises and persists, determining material cycling and energy flows among species that have fundamental controlling effects on their ecological dynamics (Paine 1980; Pimm et al. 1991), has been a long discussed topic in ecology (Allesina and Tang 2012; Garcia‐Domingo and Saldaña 2007; McCann 2000). Classical theoretical work has suggested that increased complexity can undermine ecological stability (May 1972), raising the issue of how complex food webs, which are commonly observed in nature, can persist. This contradiction becomes even more pronounced when considering that real ecosystems are subject to spatial and temporal variation in multiple environmental drivers, which may simultaneously affect network structure. However, numerous experimental studies, recently, have indicated that the complexity can emerge and persist over the long term with environmental gradient (Brauns et al. 2011; Danet et al. 2021; Leclerc et al. 2023; Ruiz‐Cooley et al. 2017; Sheaves et al. 2014; Sentis et al. 2017; Tunney et al. 2012; Takimoto and Post 2013; Tiberti et al. 2020). Furthermore, many complex ecological ecosystems are ubiquitous in nature (Laske et al. 2019). Therefore, the contradiction between theory and experiments has stimulated theoretical researchers to find several mechanisms explaining the maintenance of complexity in natural ecosystems (Borst et al. 2018; Gauzens et al. 2016; Kondoh 2003; Neutel et al. 2002; Yodzis 1981). There are a lot of models available to clarify trophic structures complexity, each highlighting distinct drivers that can influence the control of network structure. Many frameworks, for instance, have emphasized the significance of adaptive foraging for complex networks emergence (Guill and Drossel 2008; Heckmann et al. 2012; Kondoh 2003, 2006; Petchey et al. 2008; Thierry et al. 2011), real‐world structure (Marovac 1980; Mihaljevic 2012; Yodzis 1981), trophic interactions among species (McCann et al. 1998; Neutel et al. 2002; Schmitz 2007; Schröter et al. 2004), and species body size (Kartascheff et al. 2010; Osmond et al. 2017; Yodzis and Innes 1992).

However, many aforementioned theoretical frameworks, while insightful, primarily operate at local dynamics. Recognizing this, there has been a growing emphasis on integrating spatial dynamics into food‐web ecology. Recent works has begun to explore how ecosystem size (Calcagno et al. 2023; Pomeranz et al. 2023), disturbance (Terui and Nishijima 2019) effect food‐web stability and structure. Despite these important advances, the interactive influences of multiple environmental drivers, specifically ecosystem size, resource productivity, and disturbance, and species interactions such as competitive hierarchies or intransitive competitions which are fundamental to landscape‐scale ecology and can fundamentally alter trophic interactions and network persistence, on food‐web complexity are yet fully underdeveloped (Carraro and Ho 2025; Gravel et al. 2016; Guo, Zhao, et al. 2023; Ryser et al. 2021, 2024; Thompson et al. 2025; Thompson and Townsend 2005).

Different environmental drivers can shift the complexity of food webs via changing species distribution, richness and composition (Laske et al. 2019; Leclerc et al. 2023). Ecosystem size has frequently been showed to be an essential factor influencing community complexity by affecting occupation range (Post et al. 2000). Indeed, larger ecosystems promote higher dispersal rates and micro‐environment diversity, resulting in species‐rich trophic systems that exhibit increased complexity (Bhattacharyya and Sinha 2009; Calcagno et al. 2023; Mougi 2017; Post et al. 2000). For example, Pillai et al. (2011) extended metacommunity theory using a patch‐dynamic model to show the relationship between network complexity characterized by branching links that are supported by omnivore, generalist feeding links and ecosystem size, represented by habitat destruction. In addition, many studies suggest that trophic complexity is positively related to ecosystem size, measured as river area, lake size, habitat connectivity, and lake surface area (Bellmore et al. 2015; Hershey et al. 2006; LeCraw et al. 2014; Post 2002a). For instance, Beisner et al. (2006) revealed that with increasing spatial scale, trophic complexity increased. Interestingly, Parker and Huryn (2013) used mean summer discharge to find no relationship between stream size and complexity represented by mean food chain length. Moreover, Laske et al. (2019) implied that ecosystem size, represented by lake surface area, was not related to network complexity measured by species richness, total number of links and link density.

Resource productivity is another crucial factor of food‐web complexity, governing the basal energy input and its flow through trophic levels (Hawkins et al. 1997; Heino et al. 2015; Kondoh 2001; Takimoto and Post 2013; Thompson and Townsend 2005). The relationship, however, is not straightforward and is often contextualized by the classic “Enrichment paradox” (Rosenzweig 1971), which posits that increased resource productivity can destabilize ecosystems and simplify networks structure. Empirically, both positive relationships (Coll et al. 2011; Worm and Duffy 2003) and instances of no clear effect (Doi et al. 2009; Jake Vander Zanden and Fetzer 2007) have been observed. Notably, some studies have discovered increasing available resources actually cause shorter food chains (Arim et al. 2007; Holt and Polis 1997). This counterintuitive outcome is often explained by strong top‐down control: high resource productivity supports abundant top predators whose intense pressure can eliminate intermediate consumers, truncating the food web (Diehl and Feissel 2001). Furthermore, the effect of resource productivity is rarely isolated; it interacts intricately with other drivers such as temperature. For instance, warming can modulate the productivity‐complexity relationship by altering metabolic demands and interaction strengths, leading to complex and sometimes non‐additive outcomes for network stability and structure (Binzer et al. 2016; Bonnaffé et al. 2024; Tabi et al. 2019). This highlights the necessity of moving beyond single‐factor analyses to understand how resource productivity interacts with other environmental drivers in shaping food‐web complexity.

In addition to ecosystem size and resource productivity, the extent of disturbance can also influence food‐web complexity (Parker and Huryn 2013). Disturbance has negatively profound effects on network complexity by shaping species persistence, trophic interactions between species and ecological characteristics such as connectivity (Haynes et al. 2014; Hershey et al. 1999; Jackson et al. 2001; Merrick et al. 1992; Parker and Huryn 2013). Networks experiencing frequent disturbances (e.g., storm flows), for instance, have been evidenced to have lower complexity (e.g., a smaller species richness, shorter food chain length and fewer links per species) than those that undergo fewer disturbances (McHugh et al. 2010; Parker and Huryn 2011; Townsend et al. 1998). In contrast, trophic interaction complexity, indicated by link density and connectance, is in line with disturbance regardless of community size or freezing regime (Parker and Huryn 2006; Stanley et al. 2010).

The predominant approaches, until now, have been to explode the connections between environmental conditions and food‐web complexity by concentrating on specific complex metrics (e.g., food chain length, Ruiz‐Cooley et al. 2017; Ward and McCann 2017; Warfe et al. 2013) or a solitary environmental factor (e.g., resource productivity, Borst et al. 2018; Pillai et al. 2011). For instance, our previous work (Guo, Barabás, et al. 2023) demonstrated that resource productivity and disturbance could drive non‐monotonic changes in maximum food chain length in aquatic food webs. Concurrently, other researchers are exploring similar issues using complementary approaches, such as comparing mechanistic models with large empirical databases (Shibasaki and Terui 2024). However, these environmental drivers usually vary simultaneously, and measuring the complexity of a food web often requires multiple indicators for a given network (Montoya et al. 2009; Woodward et al. 2010). To this end, studying the interactive effects of environmental drivers on food‐web complexity is crucial in theory as it can help to predict how network function responds to external environmental change and hence help to develop relevant management strategies (Leclerc et al. 2023).

More importantly, many existing models neglect the role of species competition and dispersal limitation in mediating the response of complex trophic networks to environmental drivers (Fortuna and Bascompte 2006; Gravel et al. 2016; Li et al. 2020; Post 2002b). To address how landscape‐scale drivers (ecosystem size, resource productivity, and disturbance) influence communities, a patch‐dynamic metacommunity framework is particularly apt, as it directly links habitat patch availability, dispersal, and local interactions to emergent regional patterns (Calcagno et al. 2006; Leibold et al. 2004). Within this framework, a key principle is the colonization‐competition (C‐C) tradeoff, a classic mechanism explaining species coexistence and biodiversity patterns in spatially structured habitats (Calcagno et al. 2006; Tilman 1994). While dispersal abilities can vary across trophic levels (Albert et al. 2023; Ryser et al. 2021), we focus the C‐C tradeoff specifically on basal species for several reasons. Firstly, basal species form the resource foundation of the food web; their composition directly dictates the potential energy pathways and thus fundamentally constrains higher‐trophic‐level assembly. Secondly, in many ecosystems (e.g., plants in terrestrial habitats, phytoplankton or benthic producers in aquatic ecosystems), competition for space and light is a primary axis of interaction, making the C‐C tradeoff a parsimonious and powerful starting point to model their dynamics (Calcagno et al. 2023; Liao et al. 2022). This simplification allows us to isolate how environmentally driven changes in basal layer composition cascade upward to shape overall food‐web complexity. Furthermore, while stochastic dispersal and demographic processes are important in metacommunities, our deterministic formulation aims to reveal the core mechanisms arising from species' tradeoffs and environmental forcing. Consequently, in this study, we develop a patch‐dynamic framework incorporating C‐C tradeoffs among basal species for complex trophic networks, allowing us to examine how multiple environmental drivers (ecosystem size, resource productivity, and disturbance) interact to govern the emergence of food‐web complexity in spatially structured ecosystems.

## 2 Methods

### 2.1 Modeling Framework

We consider an ecosystem consisting of numerous discrete and identical patches. A fundamental assumption of our model is an asymmetry in how basal and consumer species interact with space. For basal species, we assume they compete for exclusive occupancy of a patch. Each patch can be occupied by at most one basal species due to the competitive displacement. For consumer species, we assume they do not compete directly for space with each other or with basal species within a patch. Multiple consumer species can co‐occur in the same patch, provided their required prey are present. Particularly, we assume that this ecosystem size is *S*, representing the total number of habitat available for species‐colonization utilization. Specifically, a superior species is assumed to can displace immediately the inferior residence between the basal species that cannot coexist stably within the same habitat patch (*competitive displacement*; cf. Tilman 1994; Tilman et al. 1994). Resource productivity (*R*) scales the colonization rate of basal species, and disturbance extent (*D*) increases the mortality rate of all species in a density‐independent manner. As such, the dynamics of patch occupancy for basal species i and consumer species *k* are governed by the following equations, which represent the final form of the model (*framework analysis* see Appendix S1).

For basal species *i*:

(1) $$\begin{array}{c} \frac{dP_{i}}{\mathrm{dt}}=\underset{\text{Colonization}}{\underbrace{{\mathrm{Rc}}_{i}^{P}P_{i}\left(S-\sum_{j=1}^{n_{P}}P_{j}\right)}}-\underset{\text{Extinction}}{\underbrace{e_{i}^{P}P_{i}}}+\underset{\text{Competitive displacement}}{\underbrace{R\sum_{j=1}^{n_{P}}\left(c_{i}^{P}P_{i}H_{\mathrm{ij}}P_{j}-c_{j}^{P}P_{j}H_{\mathrm{ji}}P_{i}\right)}}\\ \;+\underset{\text{Disturbance}}{\underbrace{P_{i}\log\left(1-D\right)}}-\underset{\text{Predation}}{\underbrace{P_{i}\sum_{k=1}^{n_{A}}\theta_{\mathrm{ik}}\mu_{\mathrm{ik}}A_{k}}} \end{array}$$

For consumer species *i*:

(2) $$\begin{array}{c} \frac{dA_{i}}{\mathrm{dt}}=\underset{\text{Colonization}}{\underbrace{c_{i}^{A}A_{i}\left(\sum_{j=1}^{n_{P}}\theta_{\mathrm{ji}}P_{j}+\sum_{k=1}^{n_{A}}\delta_{\mathrm{ki}}A_{k}\right)\left(S-A_{i}\right)}}-\underset{\text{Extinction}}{\underbrace{e_{i}^{A}A_{i}}}\\ \;+\underset{\text{Disturbance}}{\underbrace{A_{i}\log\left(1-D\right)}}-\underset{\text{Predation}}{\underbrace{A_{i}\sum_{k=1}^{n_{A}}\delta_{\mathrm{ik}}\varphi_{\mathrm{ik}}A_{k}}} \end{array}$$

where $P_{i}$ and $A_{k}$ indicate the site occupancy of basal species i and consumer k separately, $c_{i}^{P}$ represent the colonization rates of basal species i. $e_{i}^{P}$ and $\mu_{\mathrm{ik}}$ separately represent mortality rate and top‐down predation extinction rate of basal species i. The number of basal species i and consumer k are $n_{P}$ and $n_{A}$ respectively.

$H_{\mathrm{ij}}$ is the competition strength of basal species i contrasted to basal species j. The connectivity matrix between basal species i and consumer k is $\theta_{\mathrm{ik}}$. $\theta_{\mathrm{ik}}$ = 1 expresses that consumer k feeds on basal species i, otherwise $\theta_{\mathrm{ik}}$ = 0. S, R and D respectively show ecosystem size, resource productivity and disturbance extent.

$c_{i}^{A}$, $e_{i}^{A}$ and $\varphi_{\mathrm{ik}}$ are separately colonization rate, intrinsic mortality and top‐down predation rate of consumer i. $\delta_{\mathrm{ki}}$ = 1 represent consumer i can feed on consumer k (otherwise $\delta_{\mathrm{ki}}$ = 0). The *colonization* term describes an increase of species i owning to their own colonization of unoccupied patches. The *extinction* term describes intrinsic mortality of basal species, while the *predation* term is the population losses of species i feeded by various consumers. The *competitive displacement* term describes colonizers from species (i or j) reach a site occupied by species (jori) and displace it, respectively (Li et al. 2020; Liao et al. 2022). Particularly, the variables $H_{\mathrm{ij}}$ and $H_{\mathrm{ji}}$ are independent probabilities that species i displaces j and species j displaces i, separately. In addition, both $H_{\mathrm{ij}}$ and $H_{\mathrm{ji}}$ can indicate a variety of competition structures, for instance, a strict hierarchical competition via setting $H_{\mathrm{ij}}$ = 1 if i < j and 0 otherwise (Tilman et al. 1994), and intransitive competition via perturbing the hierarchical competition matrix *H* (Rojas‐Echenique and Allesina 2011). The *disturbance* term describes as increasing disturbance, the populations of species gradually decrease.

### 2.2 Numerical Approach for Environmental Drivers of Food‐Web Complexity

These models simulate temporal changes in species presence‐absence to investigate the causal mechanisms through which environmental gradients shape trophic network structure. We use four metrics to quantify different dimensions of food‐web complexity. Species richness (*N*) is the total number of species persisting in the food web at equilibrium. Connectance (*C*) measures the realized proportion of possible trophic links, quantified as *C* = *L*/*N* ^2^, where *L* is the number of realized prey–predator interactions. Omnivory (*O*) is defined as the fraction of consumer species that feed on prey from more than one trophic level. Mean food chain length (*MFCL*) is computed as the arithmetic mean of food chain lengths, which the food chain length is calculated using an energy‐based food web approach (Zhang et al. 2013). Then, we establish a colonization‐competition (C‐C) trade‐off among basal species by assigning them a rank‐order based on their colonization rates (from species 1 with the lowest to species $n_{P}$ with the highest, i.e., $c_{1}^{P}<c_{2}^{P}<\cdots <c_{n_{P}}^{P}$) and ranking the species competition strength in a strict hierarchical competition from the best competitor (species 1) to the poorest (species $n_{P}$). In conclusion, colonization rates of basal species are negatively correlated with competition ability in the C‐C trade‐off within the food web (Tilman 1994). Firstly, we analyze how these environmental drivers affect ecosystem complexity in some empirical food webs. Additionally, we offer a causal mechanism for results by basal species diversity along these environmental factors. Lastly, we demonstrate the robustness of the resulting food‐web structures via using 100 complex food webs.

To generate realistic food‐web structures for our simulations, we utilized the niche model (Williams and Martinez 2000). This framework is widely used in theoretical food‐web ecology because it can generate networks that closely resemble real network structures and requires only two parameters (the total number of species *N* and connectance *C*). The alternative models that are designed to be acyclic by construction, such as the generalized cascade model (Stouffer et al. 2005) and modified versions of the niche model (Johnson et al. 2014), but we still chose the classic niche model for its parsimony, historical precedence, and its established ability to generate structurally realistic food webs.

Since trophic loops can complicate the calculation of trophic levels and food chain lengths, we post‐process all generated food webs to remove any instances of loops, cannibalism and the number of basal species $n_{P}\leq 2$ in the food webs. The initial total species number was drawn randomly from $N\in \left[10,50\right]$, with a mean $\bar{N}=30$, a standard deviation SD=10, and the initial connectance was drawn randomly from $C\in \left[0.05,0.25\right]$, with a mean $\bar{C}=0.15$, a standard deviation SD=0.05, by analyzing relevant datasets from real food webs (Digel et al. 2011). The initial food webs in this study, therefore, can capture the characteristics of natural communities.

### 2.3 Intransitive Competition

The intransitive degree can be calculated using the relative intransitivity (*RI*) index of the competition matrix *H* following previous works (Laird and Schamp 2006, 2008; Li et al. 2020; Rojas‐Echenique and Allesina 2011), with $\mathrm{RI}=1-\frac{{\mathrm{Var}}_{\mathrm{obs}}-{\mathrm{Var}}_{\min}}{{\mathrm{Var}}_{\max}-{\mathrm{Var}}_{\min}}$. Here ${\mathrm{Var}}_{\mathrm{obs}}$ denotes the variance of the row sums by using the competition matrix *H*. ${\mathrm{Var}}_{\max}$ and ${\mathrm{Var}}_{\min}$ are, respectively, the maximum and minimum possible variances for the row sums of a competitive tournament matrix with the same number of species as the observed tournament matrix. The degree of intransitivity (*RI*) interacts with C‐C tradeoff to modulate how environmental drivers filter the basal species pool. When ${\mathrm{Var}}_{\mathrm{obs}}$ equals ${\mathrm{Var}}_{\min}$, *RI* = 1; while ${\mathrm{Var}}_{\mathrm{obs}}$ is equals to ${\mathrm{Var}}_{\max}$, *RI* = 0. Environmental drivers (e.g., disturbance *D*, resource productivity *R*) alter the effective growth rates of all basal species. Changes can shift the outcome of these intransitive loops, thereby altering which subset of basal species persists under a given environmental condition. This, in turn, reconfigures the supporting resource base for the entire consumer network. In this paper, therefore, we primarily generate three levels of *RI* (0, 0.5 and 1) for basal species competition in the complex networks to find how environmental factors affect food‐web complexity.

### 2.4 Simulations

We initially simulate each scenario until the system reaches dynamical equilibrium. Based on extensive preliminary simulations, we determine that a duration of 140,000 time units is sufficient for all cases to stabilize. To ensure robustness, each simulation was extended to 150,000 time units. Species abundances are computed as the mean site occupancy over the final 5000 time units. Numeric solutions for patch occupancy dynamics are obtained using the ODE45 solver in MATLAB R2020b. Food‐web complexity is estimated from these steady‐state occupancies, with a species considered extinct if its abundance fell below a threshold of 10^−6^ at equilibrium. Moreover, all numerical simulations and results presented in this study are based on the full model described by Equations (1) and (2), which includes top‐down predation pressure on both basal and intermediate consumer species.

In addition, the framework incorporates several key mechanisms that can control food‐web complexity. Firstly, environmental drivers and C‐C tradeoffs determine which basal species coexist, setting the initial energy channels, that is, bottom‐up control via basal layer composition. Secondly, predation pressure can control prey populations, with effects that cascade through food web, namely, top‐down control on basal and intermediate species. Moreover, C‐C tradeoff drives species sorting among patches and disturbance *D* affects all species equally, including basal species and consumers, providing a neutral filter.

## 3 Results

We first present the interactive and individual effects of ecosystem size (*S*), resource productivity (*R*), and disturbance (*D*) on complexity metrics, species richness (*N*), connectance (*C*), omnivory (*O*), and mean food chain length (*MFCL*) for a representative food web. We then explore the underlying mechanism by examining the basal species dynamics, and finally demonstrate the robustness of the results across a wide range of network structures and competition cases.

**FIGURE 1:**
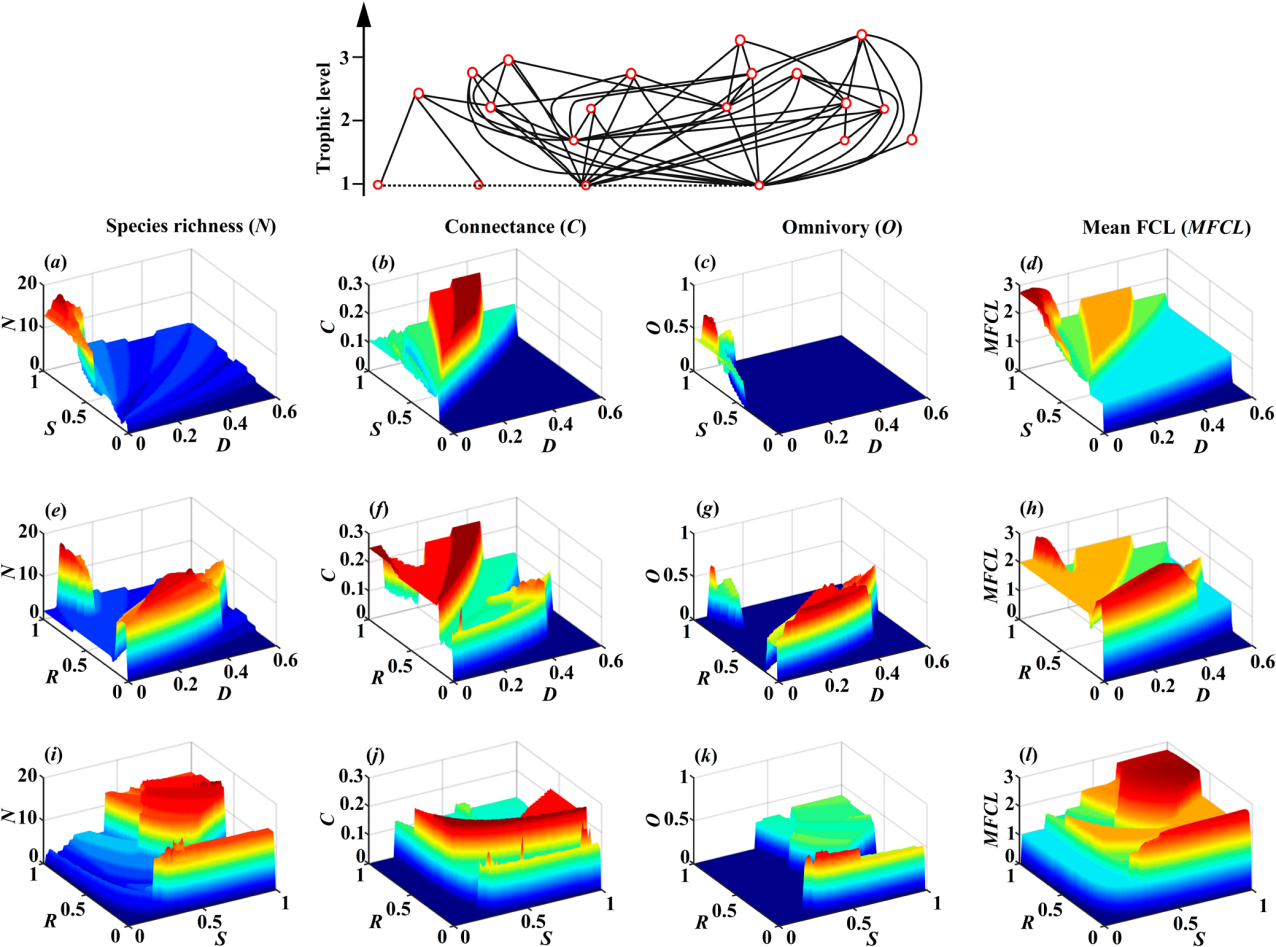
Interactive effects of ecosystem size (*S*), resource productivity (*R*) and disturbance extent (*D*) on the food‐web complexity, indicated by species richness (*N*), connectance (*C*), omnivory (*O*) and mean food chain length (*MFCL*). The initial total number of species, connectance and the initial number of basal species are 20, 0.15 and 4, respectively. The red circles represent species and the black lines show trophic links, while the dotted lines illustrate competition between basal species. A strict hierarchical competition ($H_{\mathrm{ij}}=1$ for i<j and 0 otherwise in a matrix *H*) is considered by ranking the species competition ability from the best competitor (species 1) to the poorest (species $n_{P}$). The colonization rates of basal species are ranked in increasing order at $c_{i}^{P}\in E\left[0.5,4\right]$, while all consumers colonization rates are $c_{i}^{A}=2.25$. The extinction rates of both basal species and consumers are $e_{i}^{P}=e_{i}^{A}=0.05$, and all top‐down extinction rates due to predation are equal with $\mu_{\mathrm{ik}}=\varphi_{\mathrm{ik}}=0.05$. Other parameters: *R* = 1 in *D*‐*S* interactive effects (panels a–d), *S* = 1 in *D*‐*R* interactive effects (panels e–h), and *D* = 0 in *R*‐*S* interactive effects (panels i–l).

The interactive effects of ecosystem size, resource productivity, and disturbance on food‐web complexity are shown in Figure 1. All four food‐web complexity characteristics exhibit pronounced, non‐linear idiosyncrasy along the gradients of any two environmental drivers while the third is held constant. Notably, the idiosyncratic relationships are of great amplitude and show frequent shifts between peaks and troughs. This indicates that the quantitative architecture of the network (link density and distribution), the sheer number of species, and the average trophic level are sensitive to environmental interactions. The observed multimodality suggests that a given combination of environmental conditions can lead to multiple distinct levels of food‐web complexity, driven by changes in the underlying species composition.

Then, we analyzed the influence of environmental factors on food‐web complexity using three typical food webs (with basal species $n_{P}$ = 3, 5 and 6). We observed that the network structures consistently exhibited idiosyncratic dynamics under environmental variation, mirroring the pattern shown in Figure 1 (Figures S1–S3). Moreover, we explored the effects of environmental factor interactions on food‐web complexity using a highly complex, model‐generated food web (with basal species $n_{P}$ = 10, total species richness *N* = 41, and connectance *C* = 0.15). The food web structures are idiosyncratic changes under various environmental factors (Figure S4). This highlights that the relationship between environmental drivers and food‐web architecture is highly context‐dependent and cannot be captured by simple linear or unimodal models.

**FIGURE 2:**
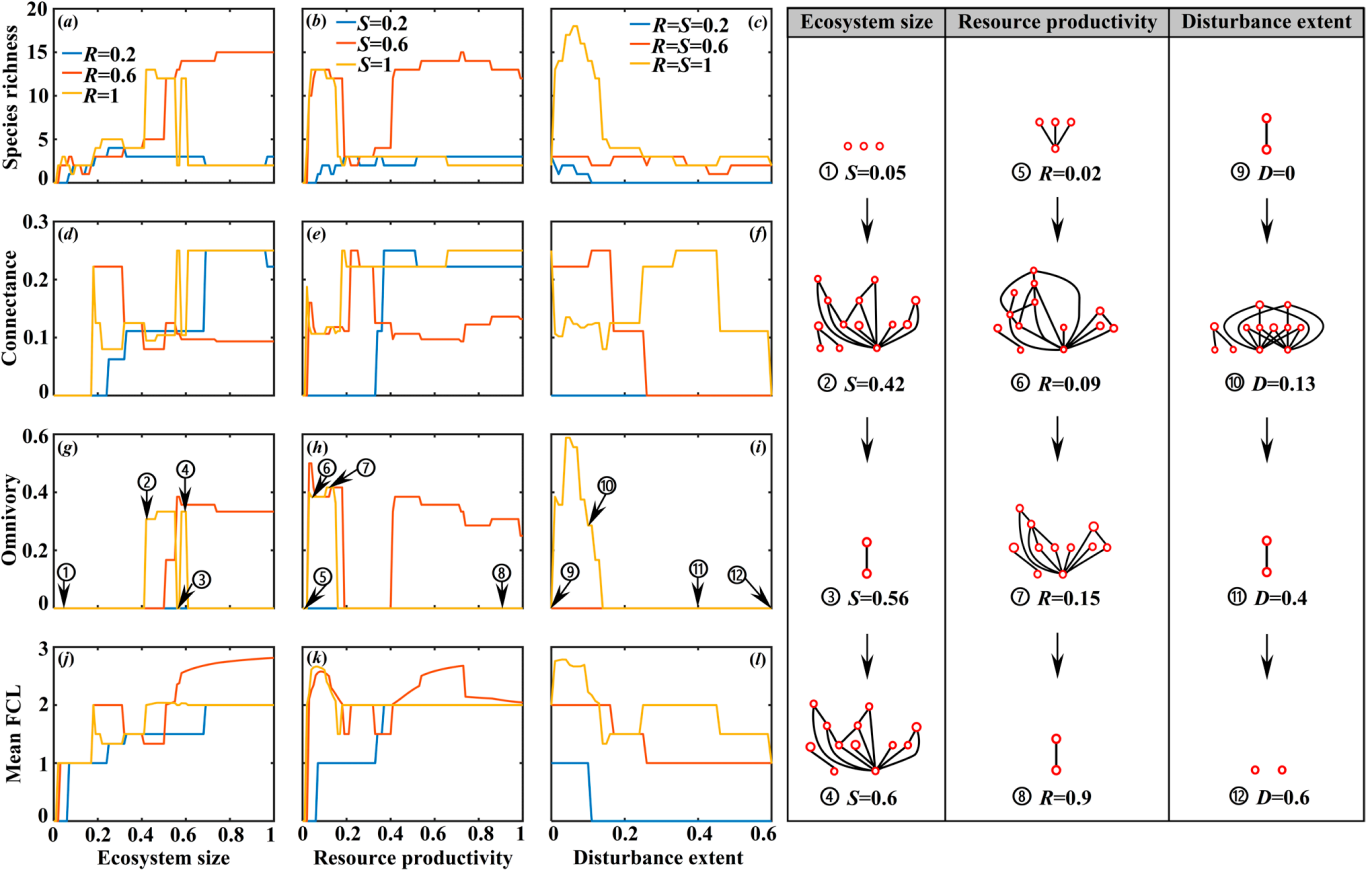
Individual effects of ecosystem size (*S*), resource productivity (*R*) and disturbance extent (*D*) on the food‐web complexity (characterized by species richness, connectance, omnivory and mean FCL). *R* = 0.2, 0.6 and 1 with *D* = 0 in panels (a, d, g, j); *S* = 0.2, 0.6 and 1 with *D* = 0 in panels (b, e, h, k); *S* = *R* = 0.2, 0.6 and 1 in panels (c, f, i, l). At the same time, four network structures are exhibited for each environmental driver. Other parameter settings and the initial food web are the same as in Figure 1.

The individual effects of each environmental driver on food‐web complexity reveal similar response patterns (Figure 2). Species richness, connectance, omnivory, and Mean food chain length displayed highly non‐monotonic, often multimodal relationships with all three environmental drivers. This underscores that a richer community does not necessarily imply a more connected or omnivorous one. For each environmental factor, four specific food webs were selected to demonstrate on the right panels of Figure 2, visually confirming that the food web does not become continuously more complex or simpler, but rather exhibits complex variations along each gradient.

**FIGURE 3:**
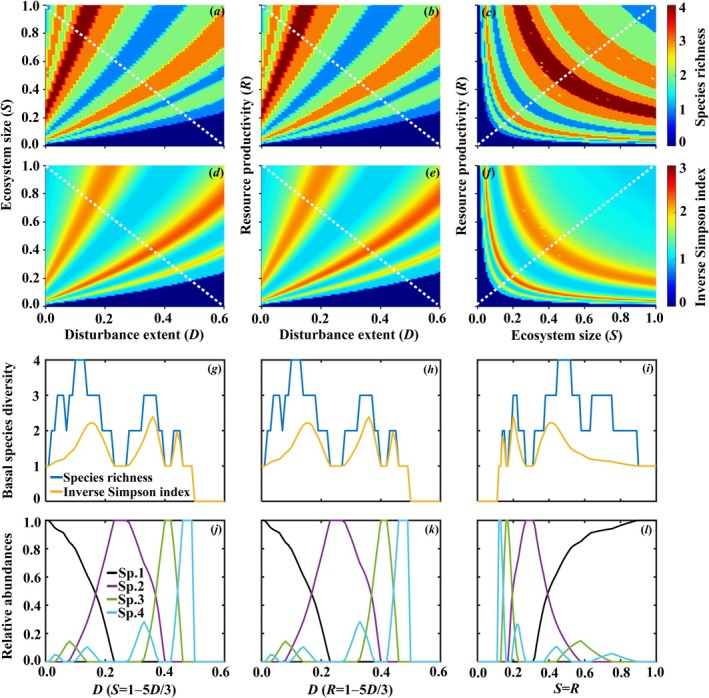
Interactive effects of ecosystem size (*S*), resource productivity (*R*) and disturbance extent (*D*) on basal species complexity which is indicated by species richness (a–c) and inverse Simpson index (d–f) for initial species richness $n_{P}$ = 4, while ignoring the top‐down predation. The inverse Simpson index is calculated by 1∑qi2 (qi=Pi∑Pj being the relative abundance of basal species i). Meanwhile, the white dotted lines in interactive effects are displayed by using basal species diversity (g–i) and their relative abundances (j–l). Other parameters are the same as in Figure 1.

To explain food‐web complexity mentioned above, we considered the interaction between ecosystem size, resource productivity and disturbance extent on the biodiversity of basal species (characterized by species richness and inverse Simpson index) without predation pressure. The inverse Simpson index is calculated by 1∑qi2 (qi=Pi∑Pj being the relative abundance of basal species i). The interactive effects show idiosyncrasy (see Figure 3). In particular, the non‐monotonicity in species richness of basal species is evident. Furthermore, we presented the species richness, inverse Simpson index and their relative abundances along the diagonal or off‐diagonal intersections (represented by white dotted lines in Figure 3). The biodiversity of basal species consistently displays idiosyncratic patterns (Figure 3g–i). The relative abundances of each species also exhibit non‐monotonicity (Figure 3j–l). As basal species changed under the environmental factors, the consumers supported by these basal species differed, resulting in different food webs under different combinations of environmental conditions. Therefore, the food web adapts accordingly, exhibiting complexity under the environmental drivers. Similarly, Figures S5–S7 show cases with the basal species of 3, 5 and 6, respectively.

**FIGURE 4:**
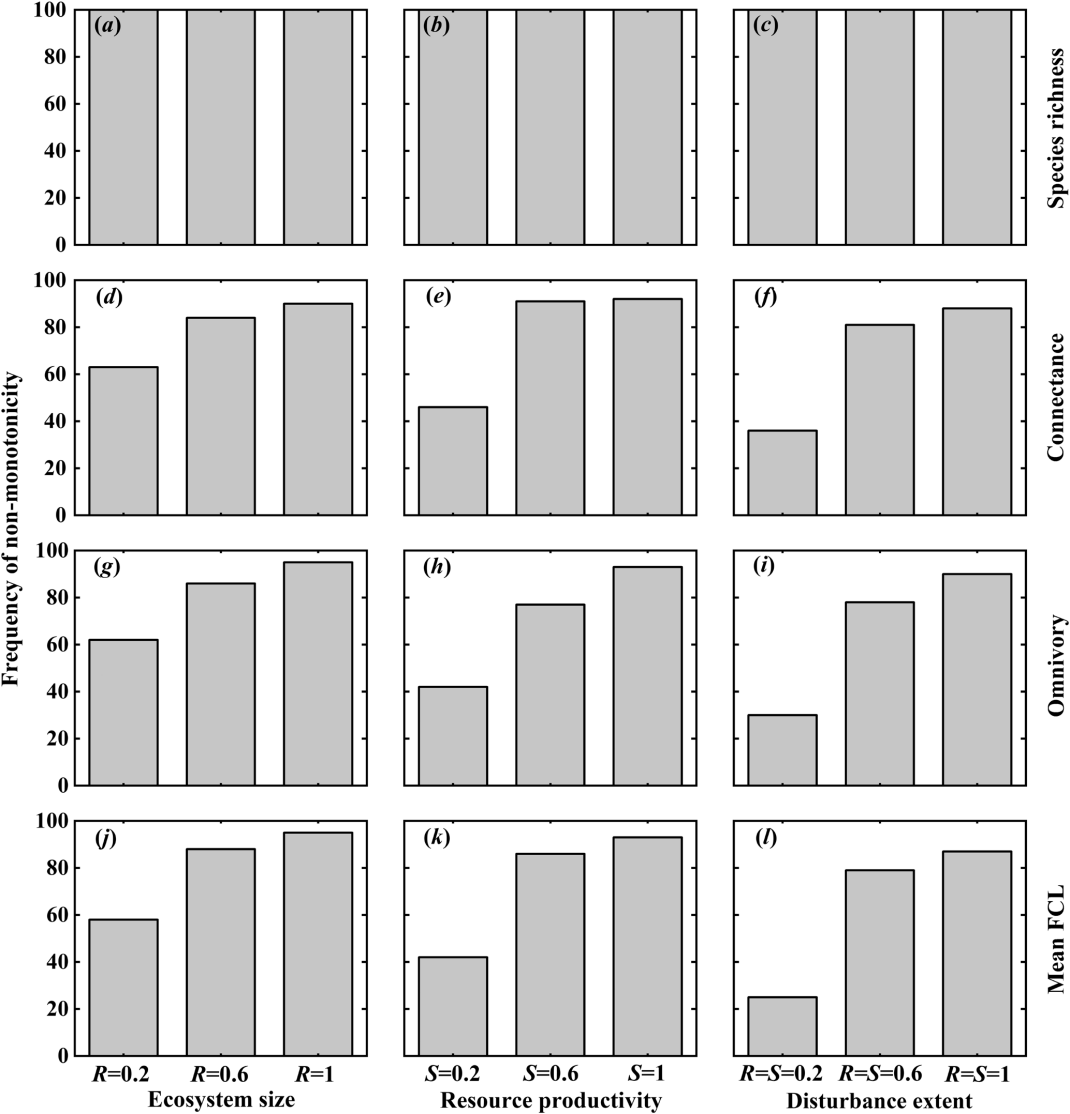
Frequency of the non‐monotonic relationship between network complexity (showed by species richness, connectance, omnivory and mean FCL) and each environmental factor, which include ecosystem size (*S*), resource productivity (*R*) and disturbance extent (*D*) in 100 initial complex food webs (no loops, no cannibalism and the number of basal species $n_{P}\geq 3$) generated by niche model. The species richness of these food webs varies from 10 to 50 and the connectance varies from 0.05 to 0.25, while the minimum number of basal species is three. *R* = 0.2, 0.6 and 1 with *D* = 0 in panels (a, d, g, j); *S* = 0.2, 0.6 and 1 with *D* = 0 in panels (b, e, h, k); *S* = *R* = 0.2, 0.6 and 1 in panels (c, f, i, l). Other parameters see Figure 1.

We examined the non‐monotonicity (representing the food‐web complexity) of species richness, connectance, omnivory, and mean food chain length along ecosystem size, resource productivity, and disturbance extent increase by using 100 initial food webs generated by niche model to demonstrate the robustness of above results (Figure 4). Species richness exhibited non‐monotonic changes in 100% of food webs across all environmental gradients (Figure 4a–c). For connectance, omnivory, and mean food chain length, the frequency of non‐monotonic food webs remained high (typically > 80%) but showed more context dependence. This hierarchy, from universally idiosyncratic species richness to conditionally idiosyncratic network structure, highlights that different facets of food‐web complexity are decoupled and respond with different sensitivities to environmental change.

In addition, we analyzed the response of food web structural characteristics (species richness, connectance, omnivory and mean food chain length) to environmental factors using five complex food‐web topologies generated by the niche model. We compared two cases: weakening the competitive hierarchy *H* of basal species (Figures S8–S12) vs. drawing uniformly the colonization rates of basal species (Figures S13–S17). The food web structures are idiosyncratic in response to environmental factors, particularly when colonization rates of basal species are randomly varied. This indicates that environmental factors drive the food‐web complexity. We also counted the number of non‐monotonic food webs among 100 food webs under these two scenarios (Figures S19 and S20). We find that, in most cases, the number of non‐monotonic food webs exceeds 80, indicating that the food web characteristics are all oscillating. Furthermore, we used a real food web (see Figure 1) to analyze the idiosyncratic driving by ecosystem size, resource productivity and disturbance extent when competition strength among basal species was weakened. The food web structures are, as well, idiosyncratic in all competitive cases. Additionally, the idiosyncratic, often multimodal relationships become gradually weakened as the competition strength among basal species decreases (Figure S18).

Up to now, we have operated numerical simulations in a fully competitive hierarchy among basal species (a better competitive species usually wins against the weaker species, with *RI* = 0), evenly weakening the competition strength of basal species, a small or large number of basal species ($n_{P}=3,4,5,6,10$) and 100 random food webs. However, the outcomes we predicted are robust to relaxing these cases. Meanwhile, we examined two other competitive intransitivity *RI* = 0.5 (Figures S21–S24) and *RI* = 1 (Figures S25–S29). The food webs still are idiosyncratic in reaction to environmental variables under intransitive competition among basal species irrelevant to the number of basal species. We also evaluated the amount of idiosyncratic food webs among 100 random food webs created by niche model along ecosystem size, resource productivity and disturbance extent gradually increase (Figures S30 and S31). With a few exceptions, the majority of food webs present idiosyncratic behavior (over 80%). Significantly, the volume of idiosyncratic food webs exceeded 80 for species richness in all cases, indicating that food webs are complex under the environmental drivers. These results once again confirm the robustness of our predictions, demonstrating that food‐web complexity emerges driving by the environmental factors.

## 4 Discussion

It is worth emphasizing that environmental factors can influence the structural characteristics and complexity of food webs. Existing works primarily focus on investigating the impact of individual environmental factors on food web structure, with limited research considering the interactive effects on food‐web complexity (Borst et al. 2018; Laske et al. 2019; Leclerc et al. 2023). Specifically, most published studies have approached the topic from an experimental perspective, observing how a certain characteristic (such as biodiversity) of a food web in a given ecosystem (e.g., lake food web) changes with varying environmental factors (Gauzens et al. 2016; Guill and Drossel 2008; Hawkins et al. 1997). For example, Nauta et al. (2023) revealed biodegradable artificial reefs can improve food‐web complexity (characterized by species richness, link density and connectance) through field observation of 2.5 years in an intertidal soft‐sediment ecosystem.

Existing works, including our own previous model (Guo, Barabás, et al. 2023), have established that environmental gradients can generate complex, non‐monotonic responses in food‐web properties. For example, Shibasaki and Terui (2024) employed a comparative approach between model predictions and empirical data to assess how environmental drivers affect species richness, food chain length, and omnivory. Our study complements such efforts by providing a focused, mechanistic investigation using a spatially explicit patch‐dynamic model, allowing us to directly manipulate drivers and track the causal chain from C‐C tradeoffs and basal species shifts to the reassembly of entire consumer networks. This process‐oriented view reveals that the idiosyncratic relationships we observe are not noise but the signature of sequential shifts in the dominant basal species and their supported consumer cohorts along environmental gradients (Figure 3). Thus, our work deepens previous findings by elucidating the precise assembly mechanism that links multi‐driver environments to multidimensional food‐web complexity.

In this work, non‐monotonic responses in food‐web complexity emerge along environmental gradients due to C‐C tradeoff between basal species. Importantly, the outcomes are robust across various assumptions and parameter settings, further confirming the significant role of the environment in shaping food web structure (Post 2002b). Specifically, when species are in a favorable environment (i.e., low disturbance extent, abundant resources and large ecosystem), competitively dominant species with low colonization rates gain an advantage and have the highest species abundances. They outcompete other species and reduce the food web to a single basal species. As the environment deteriorates (i.e., increased disturbance, reduced available resources and smaller habitat), the abundance of strong competitors decreases, while species with high colonization rates become more abundant, leading to the presence of multiple basal species. With further degradation of the environment, strong competitive species no longer have an advantage, resulting in a further decrease in their abundance until they extinct. Meanwhile, species having colonization‐rates advantage are absolute dominance, manifesting an increased abundance that is modulated by predatory pressure (Figure 3j–l). Species with intermediate competitive abilities and colonization rates fluctuate in their presence and absence due to consumer predation and competition exclusion, resulting in different subsets of basal species during environmental changes. Each subset of basal species supports different consumers, therefore, leading to distinct food webs composed of basal species and their associated consumers.

Additionally, idiosyncratic relationships in food‐web complexity can only be seen when the potential food web is sufficiently complex; otherwise, such phenomenon cannot be viewed. Each consumer must have lower trophic species as their food sources; when a particular species at lower trophic level dies, the consumer can still survive by preying on other species. When the food web is in a favorable environment and is potentially complex enough, the omnivory of the food web is relatively high, and more species can prey on multiple species at lower trophic levels, leading to observable idiosyncrasy in the food web (Pimm and Lawton 1977). However, in harsh environments, where the potential food web is too simple (i.e., usually with only two trophic levels), most food web structures (e.g., connectance, omnivory and mean food chain length) do not show idiosyncratic patterns under the interaction between environmental drivers and C‐C tradeoffs among basal species (Figure 2d–l). Nevertheless, the species richness of the food web portrays idiosyncrasy in either favorable environments or harsh conditions because the number of basal species is bound to change along environmental gradients (Figure 2a–c). This is also why the frequency of observed non‐monotonicity on species richness is 100% (Figure 4a–c).

Our findings resonate with but also extend the growing body of theory on environmental drivers of food webs. Compared to models that focus on individual environmental driver effects or single food‐web complexity metrics, our framework explicitly tests for interactions among three ubiquitous drivers. Unlike our published work (Guo, Barabás, et al. 2023), which assumed that all consumer species are unaffected by disturbances and only analyzed a single food‐web complexity metric, the maximum food chain length specifically focuses on aquatic food webs. Therefore, the specific parameter values selected for numerical simulations also differ. Moreover, ecosystem size exerted a monotonic influence on food chain length, in contrast to the idiosyncratic effects observed in this paper.

Our focus is on spatial competition among basal species in our model and we use complex food webs instead of simple trophic modules compared with previous models (Liao et al. 2016; Parker and Huryn 2013; Townsend et al. 1998; Warfe et al. 2013). At the same time, our model notes the impact of disturbances on all species in food webs, which is closer to real ecosystems since all species including basal species and consumers are affected by external disturbances (Parker and Huryn 2013). Explicitly, the outcomes in this model are robust, requiring only the assumption of C‐C tradeoffs among basal species. Many theoretical (Guill and Drossel 2008; Guo, Zhao, et al. 2023; Parker and Huryn 2013; Pillai et al. 2011) and empirical (Laske et al. 2019; Leclerc et al. 2023; Nauta et al. 2023; Sheaves et al. 2014; Tiberti et al. 2020) studies have found how biodiversity reacts to environmental gradients. Moreover, there are some experimental cases that prove the response of biodiversity to disturbance and resource productivity (Hastings 1980; Parker and Huryn 2013), further demonstrating how the characteristics of food webs reply environmental factors in a multi‐peak fashion. These findings indirectly confirm our results, which can also be verified through experimental studies as carbon ($\delta^{13}C$) and nitrogen ($\delta^{15}N$) can be used to easily estimate trophic level of each species in food webs (Leclerc et al. 2023; Thompson and Townsend 2005; Zanden and Rasmussen 1999), allowing for the construction of food webs and the observation of their trophic characteristics.

The idiosyncratic responses of food‐web complexity to ecosystem size, resource productivity and disturbance extent suggest that the errors considered in experimental studies (i.e., datasets sampling errors, data processing errors, and outliers) may actually be genuine features of food webs. In other words, these values of food web characteristics exist in response to these environmental drivers and cannot be simply excluded in experimental research. Based on the findings of this study, even minor environmental changes can lead to significant shifts in food web structures including species richness, connectance, omnivory and mean food chain length. If these changes happen to occur at a critical threshold, the food web could suddenly collapse, resulting in the extinction of all species. Consequently, when formulating conservation strategies and measures, it is insufficient to solely focus on increasing habitat size or reducing various disturbances to the biota. On the contrary, it is essential to comprehensively evaluate the structural characteristics of complex trophic ecosystems and environmental conditions. More precisely, when considering biodiversity conservation, relying solely on species richness as the only goal is inadequate. The highest species richness does not necessarily correspond to the highest values of other food web structures (i.e., connectance, omnivory and mean food chain length). Furthermore, even slight environmental changes can lead to the extinction of all species. Therefore, our results suggest that, in order to protect complex yet long‐term stable food webs within entire ecosystems, it is crucial to prioritize the conservation and restoration of basal species that support them.

In conclusion, we offer a simple yet robust metacommunity framework to analyze how environmental drivers affect food‐web complexity through top‐down predation and C‐C tradeoffs between basal species. This study demonstrates the importance of the interaction between environmental factors and C‐C tradeoffs among basal species in shaping food‐web complexity. This theory provides an explicit principle for explaining how food‐web complexity changes with spatial scale under environmental drivers. The framework employs a spatially asymmetric assumption, which basal species engage in competitive exclusion for patches, while consumers can freely co‐occur within a patch. This case is particularly suited for modeling ecosystems where basal species are sessile and space‐limited (e.g., plants), while mobile consumers forage across the landscape (e.g., herbivores). Future research could potentially establish a new mechanism that combines horizontal competition and vertical interaction to explain how food‐web complexity respond to environmental factors. Additionally, it may be also possible to comprehensively analyze how food‐web complexity changes with environmental gradients via natural or laboratory experimental observations. Overall, we provide a mechanistic explanation for the emergence of food‐web complexity, further enriching our understanding of the relationships between multiple environmental drivers and food‐web complexity in complex trophic systems.

## Author Contributions

**Guanming Guo:** conceptualization (lead), data curation (lead), formal analysis (equal), funding acquisition (equal), methodology (lead), writing – original draft (lead), writing – review and editing (equal). **Helin Zhang:** formal analysis (equal), methodology (equal), writing – review and editing (equal).

## Funding

This work was supported by the National Natural Science Foundation of China (32401282); Open Fund of Key Laboratory of Low Altitude Geographic Information and Air Route of Jiangxi Education Institutes (2025LAGIAR002); Open Fund of Ministry of Education Key Laboratory for Transboundary Ecosecurity of Southwest China (YNUECO2024002).

## Conflicts of Interest

The authors declare no conflicts of interest.

## Supporting information


**Appendix S1:** ece373138‐sup‐0001‐AppendixS1.docx.

## Data Availability

The simulation codes used to run the models in this study are archived in a public repository accessible at Zenodo: https://doi.org/10.5281/zenodo.18243243.
