# Supplementary material for: A Mechanism of Food‐Web Complexity Emergence Under Multiple Environmental Drivers
Source: Ecol Evol. 2026 Feb 20;16(2):e73138. doi: 10.1002/ece3.73138 (PMC12928127; doi:10.1002/ece3.73138)
Supplement: Supplementary file 1 — Appendix S1: ece373138‐sup‐0001‐AppendixS1.docx. [file ECE3-16-e73138-s001.docx]

Supporting Information for

**A mechanism of food-web complexity emergence under multiple environmental drivers**

Guanming Guo^*^, Helin Zhang

*To whom correspondence should be addressed. Email: [ggming1990@163.com](mailto:ggming1990@163.com)

**This file includes:**

Appendix S1 – *framework analysis*

Appendix S2 – *Figures S1-S31*

**Appendix S1 – *framework analysis***

Based on previous models (Kondoh, 2001; Svensson et al., 2012), we hypothesize that basal species patch occupation is given by resource productivity using a unitless driver *R.* As such, we can write node-occupancy dynamics of basal species $i$ supported by published works (Guo et al., 2023; Hastings, 1980; Li et al., 2020; Liao et al., 2022; Tilman et al., 1994, 1997).

$\frac{dP_{i}}{dt}=\underset{Colonization}{\underbrace{{Rc}_{i}^{P}P_{i}\left( S-\sum_{j=1}^{n_{P}} P_{j} \right)}}-\underset{Extinction}{\underbrace{e_{i}^{P}P_{i}}}+\underset{Competitive displacement}{\underbrace{R\sum_{j=1}^{n_{P}} \left( c_{i}^{P}P_{i}H_{ij}P_{j}-c_{j}^{P}P_{j}H_{ji}P_{i} \right)}}+\underset{Disturbance}{\underbrace{P_{i}f(t,D,T)}}-\underset{Predation}{\underbrace{P_{i}\sum_{k=1}^{n_{A}} \theta_{ik}\mu_{ik}A_{k}}}$ (1)

The *disturbance* term can be dislodged the following equation (2) based on published literatures (Barabás et al., 2018; Chesson, 1994; Liao et al., 2016, 2022; Miller et al., 2021). The disturbance is characterized by disturbance extent (*D*) and frequency 1/T. Following (Liao et al., 2022), the influence of a disturbance with extent (*D*) and cyclicality (*T*) equals the effect of another disturbance with extent $D^{'}=1-\left( 1-D \right)^{\frac{1}{T}}$ and cyclicality *T*=1. Therefore, we only vary the disturbance extent (*D*) while keeping the periodicity *T*=1 in our model simulations. We, now, can rewrite equation (1) in the form of equation (3).

$P_{i}f\left( t,D,T \right)=P_{i}log\left( 1-D \right)/T$ (2)

$\frac{dP_{i}}{dt}=\underset{Colonization}{\underbrace{{Rc}_{i}^{P}P_{i}\left( S-\sum_{j=1}^{n_{P}} P_{j} \right)}}-\underset{Extinction}{\underbrace{e_{i}^{P}P_{i}}}+\underset{Competitive displacement}{\underbrace{R\sum_{j=1}^{n_{P}} \left( c_{i}^{P}P_{i}H_{ij}P_{j}-c_{j}^{P}P_{j}H_{ji}P_{i} \right)}}+\underset{Disturbance}{\underbrace{P_{i}log\left( 1-D \right)}}-\underset{Predation}{\underbrace{P_{i}\sum_{k=1}^{n_{A}} \theta_{ik}\mu_{ik}A_{k}}}$ (3)

Then, we model the node-occupancy dynamics of consumer $k$ in the complex food web. We assume that consumers can coexist within a same site by disregarding competition among them and a consumer has the same colonization rate when feeding on different prey species to simplify the model. Therefore, we write the node-occupancy dynamics of consumer $i$ as

$\frac{dA_{i}}{dt}=\underset{Colonization}{\underbrace{c_{i}^{A}A_{i}\left( \sum_{j=1}^{n_{P}} \theta_{ji}P_{j}+\sum_{k=1}^{n_{A}} \delta_{ki}A_{k} \right)\left( S-A_{i} \right)}}-\underset{Extinction}{\underbrace{e_{i}^{A}A_{i}}}+\underset{Disturbance}{\underbrace{A_{i}log\left( 1-D \right)}}-\underset{Predation}{\underbrace{A_{i}\sum_{k=1}^{n_{A}} \delta_{ik}\varphi_{ik}A_{k}}}$ (4)

**References**

Barabás, G., Andrea, R., & Stump, S. M. (2018). Chesson’s coexistence theory. *Ecological Monographs*, *88*(3), 277–303. https://doi.org/10.1002/ecm.1302

Chesson, P. (1994). Multispecies competition in variable environments. *Theoretical Population Biology*, *45*(3), 227–276. https://doi.org/10.1006/tpbi.1994.1013

Guo, G., Zhao, F., Nijs, I., & Liao, J. (2023). Colonization–competition dynamics of basal species shape food web complexity in island metacommunities. *Marine Life Science & Technology*, *5*(2), 169–177. https://doi.org/10.1007/s42995-023-00167-0

Hastings, A. (1980). Disturbance, coexistence, history, and competition for space. *Theoretical Population Biology*, *18*(3), 363–373. https://doi.org/10.1016/0040-5809(80)90059-3

Kondoh, M. (2001). Unifying the relationships of species richness to productivity and disturbance. *Proceedings of the Royal Society of London. Series B: Biological Sciences*, *268*(1464), 269–271. https://doi.org/10.1098/rspb.2000.1384

Li, Y., Bearup, D., & Liao, J. (2020). Habitat loss alters effects of intransitive higher-order competition on biodiversity: A new metapopulation framework. *Proceedings of the Royal Society B: Biological Sciences*, *287*(1940), 20201571. https://doi.org/10.1098/rspb.2020.1571

Liao, J., Barabás, G., & Bearup, D. (2022). Competition–colonization dynamics and multimodality in diversity–disturbance relationships. *Ecology*, *103*(5), e3672. https://doi.org/10.1002/ecy.3672

Liao, J., Ying, Z., Woolnough, D. A., Miller, A. D., Li, Z., & Nijs, I. (2016). Coexistence of species with different dispersal across landscapes: A critical role of spatial correlation in disturbance. *Proceedings of the Royal Society B: Biological Sciences*, *283*(1830), 20160537. https://doi.org/10.1098/rspb.2016.0537

Miller, A. D., Inamine, H., Buckling, A., Roxburgh, S. H., & Shea, K. (2021). How disturbance history alters invasion success: Biotic legacies and regime change. *Ecology Letters*, *24*(4), 687–697. https://doi.org/10.1111/ele.13685

Svensson, J. R., Lindegarth, M., Jonsson, P. R., & Pavia, H. (2012). Disturbance–diversity models: What do they really predict and how are they tested? *Proceedings of the Royal Society B: Biological Sciences*, *279*(1736), 2163–2170. https://doi.org/10.1098/rspb.2011.2620

Tilman, D., Lehman, C. L., & Yin, C. (1997). Habitat destruction, dispersal, and deterministic extinction in competitive communities. *The American Naturalist*, *149*(3), 407–435. https://doi.org/10.1086/285998

Tilman, D., May, R. M., Lehman, C. L., & Nowak, M. A. (1994). Habitat destruction and the extinction debt. *Nature*, *371*(6492), 65–66. https://doi.org/10.1038/371065a0

**Appendix S2 – *Figures S1-S31***





**Figure S1.** Interactive effects of ecosystem size (*S*), resource productivity (*R*) and disturbance extent (*D*) on the food-web complexity which is characterized by species richness (*N*), connectance (*C*), omnivory (*O*) and mean food chain length (*MFCL*) in a given typical food web as displayed (total species richness *N*=20, connectance *C*=0.15 and basal species number $n_{P}=3$, red circles – species, black lines – trophic links, and dotted lines – basal species competition). The competition ability among basal species in a strict hierarchical competition ($H_{ij}=1$for $i<j$ and 0 otherwise in a matrix ***H***) ranks from the best competitor (species 1) to the poorest (species $n_{P}$), while their colonization rates are spaced in increasing order at $c_{i}^{P}\in E\left[0.5,4\right]$. The extinction rates of both basal species and consumers are $e_{i}^{P}=e_{i}^{A}=0.05$, and all top-down extinction rates due to predation are equal with . Other parameters: $c_{i}^{A}=2.25$, *R*=1 in *D*-*S* interactive effects from (I) to (IV), *S*=1 in *D*-*R* interactive effects from (V) to (VIII), and *D*=0 in *R*-*S* interactive effects from (IX) to (XII).

**

**

**Figure S2.** Interactive effects of ecosystem size (*S*), resource productivity (*R*) and disturbance extent (*D*) on the food-web complexity, characterized by species richness (*N*), connectance (*C*), omnivory (*O*) and mean food chain length (*MFCL*) in a given typical food web as displayed. The number of basal species is five. Other parameters are the same as in Fig. S1.

**

**

**Figure S3.** Interactive effects of ecosystem size (*S*), resource productivity (*R*) and disturbance extent (*D*) on the food-web complexity, indicated by species richness (*N*), connectance (*C*), omnivory (*O*) and mean food chain length (*MFCL*) in a given typical food web as displayed. Basal species number is six. Other parameter settings are the same as in Fig. S1.

**

**

**Figure S4.** Interactive effects of ecosystem size (*S*), resource productivity (*R*) and disturbance extent (*D*) on the food-web complexity, showed by species richness (*N*), connectance (*C*), omnivory (*O*) and mean food chain length (*MFCL*) in a given typical food web (with species diversity *N*=41 and basal species $n_{P}=10$) as displayed. Other parameters see Fig. S1.

**

**

**Figure S5.** Interactive effects of ecosystem size (*S*), resource productivity (*R*) and disturbance extent (*D*) on basal species complexity, characterized by species richness (I-III) and inverse Simpson index (IV-VI) for initial species richness $n_{P}$=3, while ignoring the top-down predation. The inverse Simpson index is calculated by$\frac{1}{\sum q_{i}^{2}}$ ($q_{i}=\frac{P_{i}}{\sum P_{j}}$being the relative abundance of basal species $i$). The colonization rates among basal species are ranked in increasing order at $c_{i}^{P}\in E\left[0.5,4\right]$, while their competition ability is spaced from the best competitor (species 1) to the poorest (species $n_{P}$) in a strict hierarchical competition ($H_{ij}=1$for $i<j$ and 0 otherwise in a matrix ***H***) to establish the C-C tradeoffs. The extinction rates are $e_{i}^{P}=0.05$for all basal species. Meanwhile, the white dotted lines in interactive effects are displayed by using basal species diversity (VII-IX) and their relative abundances (X-XII).

**

**

**Figure S6.** Interactive effects of ecosystem size (*S*), resource productivity (*R*) and disturbance extent (*D*) on basal species complexity, indicated by species richness (I-III) and inverse Simpson index (IV-VI) for initial species richness $n_{P}$=5, while ignoring the top-down predation. At the same time, the white dotted lines in interactive effects are displayed by using basal species diversity (VII-IX) and their relative abundances (X-XII). Other parameters are the same as in Fig. S5.

**

**

**Figure S7.** Interactive effects of ecosystem size (*S*), resource productivity (*R*) and disturbance extent (*D*) on basal species complexity, indicated by species richness (I-III) and inverse Simpson index (IV-VI) for initial species richness $n_{P}$=6, while ignoring the top-down predation. At the same time, the white dotted lines in interactive effects are displayed by using basal species diversity (VII-IX) and their relative abundances (X-XII). Other parameter settings are the same as in Fig. S5.

**

**

**Figure S8.** Interactive effects of ecosystem size (*S*), resource productivity (*R*) and disturbance extent (*D*) on the food-web complexity, indicated by species richness (*N*), connectance (*C*), omnivory (*O*) and mean food chain length (*MFCL*) in a given typical food web (basal species $n_{P}=3$) as exhibited. The colonization rates among basal species are ranked in increasing order at $c_{i}^{P}\in E\left[0.5,4\right]$, while weakening their competitive hierarchy ***H***: the lower and upper triangular entries (*H_ij_*) are uniformly sampled from $U\left[0,0.25\right]$ and $U\left[0.75,1\right]$, respectively. Other parameters see Fig. S1.

**

**

**Figure S9.** Interactive effects of ecosystem size (*S*), resource productivity (*R*) and disturbance extent (*D*) on the food-web complexity, which characterized by species richness (*N*), connectance (*C*), omnivory (*O*) and mean food chain length (*MFCL*) in a given typical food web (basal species $n_{P}=4$) as showed. Other parameter settings are the same as in Fig. S8.

**

**

**Figure S10.** Interactive effects of ecosystem size (*S*), resource productivity (*R*) and disturbance extent (*D*) on the food-web complexity, which characterized by species richness (*N*), connectance (*C*), omnivory (*O*) and mean food chain length (*MFCL*) in a given typical food web (basal species $n_{P}=5$) as showed. Other parameters are the same as in Fig. S8.

**

**

**Figure S11.** Interactive effects of ecosystem size (*S*), resource productivity (*R*) and disturbance extent (*D*) on the food-web complexity, which characterized by species richness (*N*), connectance (*C*), omnivory (*O*) and mean food chain length (*MFCL*) in a given typical food web (basal species $n_{P}=6$) as showed. Other parameter settings are the same as in Fig. S8.

**

**

**Figure S12.** Interactive effects of ecosystem size (*S*), resource productivity (*R*) and disturbance extent (*D*) on the food-web complexity, which characterized by species richness (*N*), connectance (*C*), omnivory (*O*) and mean food chain length (*MFCL*) in a given typical food web (total species number *N*=41 and basal species $n_{P}=10$) as exhibited. Other parameter settings see Fig. S8.

**

**

**Figure S13.** Interactive effects of ecosystem size (*S*), resource productivity (*R*) and disturbance extent (*D*) on the food-web complexity, indicated by species richness (*N*), connectance (*C*), omnivory (*O*) and mean food chain length (*MFCL*) in a given typical food web (basal species $n_{P}=3$) as exhibited. The colonization rates among basal species are uniformly drawn from $c_{i}^{P}\in U\left[0.5,4\right]$ and sorted in increasing order, while with a strict competitive hierarchy ***H*** ($H_{ij}=1$ for $i<j$ and 0 otherwise). Other parameters are the same as in Fig. S1.

**

**

**Figure S14.** Interactive effects of ecosystem size (*S*), resource productivity (*R*) and disturbance extent (*D*) on the food-web complexity, indicated by species richness (*N*), connectance (*C*), omnivory (*O*) and mean food chain length (*MFCL*) in a given typical food web (basal species $n_{P}=4$) as exhibited. Other parameters are the same as in Fig. S13.

**

**

**Figure S15.** Interactive effects of ecosystem size (*S*), resource productivity (*R*) and disturbance extent (*D*) on the food-web complexity, characterized by species richness (*N*), connectance (*C*), omnivory (*O*) and mean food chain length (*MFCL*) in a given typical food web (basal species $n_{P}=5$) as exhibited. Other parameters are the same as in Fig. S13.

**

**

**Figure S16.** Interactive effects of ecosystem size (*S*), resource productivity (*R*) and disturbance extent (*D*) on the food-web complexity, characterized by species richness (*N*), connectance (*C*), omnivory (*O*) and mean food chain length (*MFCL*) in a given typical food web (basal species $n_{P}=6$) as exhibited. Other parameters are the same as in Fig. S13.

**

**

**Figure S17.** Interactive effects of ecosystem size (*S*), resource productivity (*R*) and disturbance extent (*D*) on the food-web complexity, characterized by species richness (*N*), connectance (*C*), omnivory (*O*) and mean food chain length (*MFCL*) in a given typical food web (species diversity *N*=41 and basal species $n_{P}=10$) as exhibited. Other parameters are the same as in Fig. S13.

**

**

**Figure S18.** Individual effects of ecosystem size (*S*), resource productivity (*R*) and disturbance extent (*D*) on the food-web complexity, characterized by species richness (*N*), connectance (*C*), omnivory (*O*) and mean food chain length (*MFCL*) in a given food web as exhibited in Fig. 1. *R*=1 and *D*=0 in panels (I, IV, VII, and X); *S*=1 and *D*=0 in panels (II, V, VIII, and XI); *S*=*R*=1 in panels (III, VI IX, and XII). The colonization rates of basal species are ranked in increasing order at $c_{i}^{P}\in E\left[0.5,4\right]$, while all consumers colonization rates are $c_{i}^{A}=2.25$. Meanwhile, we gradually weaken a strict competitive hierarchy ***H***: the upper triangular entries $H_{ij}$=1, 0.8 or 0.6, corresponding to the lower triangular entries $H_{ji}$=0, 0.2 or 0.4. The extinction rates of both basal species and consumers are $e_{i}^{P}=e_{i}^{A}=0.05$, and all top-down extinction rates due to predation are equal with .

**

**

**Figure S19.** Frequency of the non-monotonic relationship between network complexity (showed by species richness, connectance, omnivory and mean FCL) and each environmental factor, which include ecosystem size (*S*), resource productivity (*R*) and disturbance extent (*D*) in 100 initial complex food webs (no loops and cannibalism) generated by niche model. The competition ability among basal species is spaced by weakening their competitive hierarchy ***H***: the lower and upper triangular entries (*H_ij_*) are uniformly sampled from $U\left[0,0.25\right]$ and $U\left[0.75,1\right]$, respectively. Other parameter settings are the same as in Fig. 4 in the main text.

**

**

**Figure S20.** Frequency of the non-monotonic relationship between network complexity (showed by species richness, connectance, omnivory and mean FCL) and each environmental factor, which include ecosystem size (*S*), resource productivity (*R*) and disturbance extent (*D*) in 100 initial complex food webs (no loops and cannibalism) generated by niche model. The colonization rates among basal species are uniformly drawn from $c_{i}^{P}\in U\left[0.5,4\right]$ and sorted in increasing order, while with a strict competitive hierarchy ***H*** ($H_{ij}=1$ for $i<j$ and 0 otherwise). Other parameters are the same as in Fig. S19.

**

**

**Figure S21.** Interactive effects of ecosystem size (*S*), resource productivity (*R*) and disturbance extent (*D*) on the food-web complexity which is indicated by species richness (*N*), connectance (*C*), omnivory (*O*) and mean food chain length (*MFCL*) in a given typical food web as displayed (basal species number $n_{P}=4$). The colonization rates among basal species are spaced in increasing order at $c_{i}^{P}\in E\left[0.5,4\right]$, while their competitive intransitivity $RI=0.5$ ($H_{ij}=0$ or 1). Other parameters: the extinction rates of both basal species and consumers are $e_{i}^{P}=e_{i}^{A}=0.05$, and all top-down extinction rates due to predation are equal with . All consumers colonization rates are $c_{i}^{A}=2.25$. *R*=1 in *D*-*S* interactive effects from (I) to (IV), *S*=1 in *D*-*R* interactive effects from (V) to (VIII), and *D*=0 in *R*-*S* interactive effects from (IX) to (XII).

**

**

**Figure S22.** Interactive effects of ecosystem size (*S*), resource productivity (*R*) and disturbance extent (*D*) on the food-web complexity which is indicated by species richness (*N*), connectance (*C*), omnivory (*O*) and mean food chain length (*MFCL*) in a given typical food web as displayed (basal species number $n_{P}=5$). Other parameters are the same as in Fig. S21.

**

**

**Figure S23.** Interactive effects of ecosystem size (*S*), resource productivity (*R*) and disturbance extent (*D*) on the food-web complexity which is indicated by species richness (*N*), connectance (*C*), omnivory (*O*) and mean food chain length (*MFCL*) in a given typical food web as displayed (basal species number $n_{P}=6$). Other parameter settings are the same as in Fig. S21.

**

**

**Figure S24.** Interactive effects of ecosystem size (*S*), resource productivity (*R*) and disturbance extent (*D*) on the food-web complexity which is indicated by species richness (*N*), connectance (*C*), omnivory (*O*) and mean food chain length (*MFCL*) in a given typical food web as displayed (total species richness *N*=41 and basal species number $n_{P}=10$). Other parameters see Fig. S21.

**

**

**Figure S25.** Interactive effects of ecosystem size (*S*), resource productivity (*R*) and disturbance extent (*D*) on the food-web complexity which is indicated by species richness (*N*), connectance (*C*), omnivory (*O*) and mean food chain length (*MFCL*) in a given typical food web as displayed (basal species number $n_{P}=3$). The competitive intransitivity of basal species $RI=1$ ($H_{ij}=0$ or 1). Other parameter settings are the same as in Fig. S21.

**

**

**Figure S26.** Interactive effects of ecosystem size (*S*), resource productivity (*R*) and disturbance extent (*D*) on the food-web complexity which is indicated by species richness (*N*), connectance (*C*), omnivory (*O*) and mean food chain length (*MFCL*) in a given typical food web as displayed (basal species number $n_{P}=4$). Other parameters are the same as in Fig. S25.

**

**

**Figure S27.** Interactive effects of ecosystem size (*S*), resource productivity (*R*) and disturbance extent (*D*) on the food-web complexity which is indicated by species richness (*N*), connectance (*C*), omnivory (*O*) and mean food chain length (*MFCL*) in a given typical food web as displayed (basal species number $n_{P}=5$). Other parameters see Fig. S25.

**

**

**Figure S28.** Interactive effects of ecosystem size (*S*), resource productivity (*R*) and disturbance extent (*D*) on the food-web complexity which is indicated by species richness (*N*), connectance (*C*), omnivory (*O*) and mean food chain length (*MFCL*) in a given typical food web as displayed (basal species number $n_{P}=6$). Other parameters see Fig. S25.

**

**

**Figure S29.** Interactive effects of ecosystem size (*S*), resource productivity (*R*) and disturbance extent (*D*) on the food-web complexity which is indicated by species richness (*N*), connectance (*C*), omnivory (*O*) and mean food chain length (*MFCL*) in a given typical food web as displayed (species richness *N*=41 and basal species number $n_{P}=10$). Other parameters see Fig. S25.

**

**

**Figure S30.** Frequency of the non-monotonic relationship between network complexity (showed by species richness, connectance, omnivory and mean FCL) and each environmental factor, which include ecosystem size (*S*), resource productivity (*R*) and disturbance extent (*D*) in 100 initial complex food webs (no loops, no cannibalism and the number of basal species $n_{P}\geq4$) generated by niche model. The colonization rates among basal species are spaced in increasing order at $c_{i}^{P}\in E\left[0.5,4\right]$, while their competitive intransitivity $RI=0.5$ ($H_{ij}=0$ or 1). the extinction rates of both basal species and consumers are $e_{i}^{P}=e_{i}^{A}=0.05$, and all top-down extinction rates due to predation are equal with . Other parameters: $c_{i}^{A}=2.25$, *R*=1 in *D*-*S* interactive effects from (I) to (IV), *S*=1 in *D*-*R* interactive effects from (V) to (VIII), and *D*=0 in *R*-*S* interactive effects from (IX) to (XII).

**

**

**Figure S31.** Frequency of the non-monotonic relationship between network complexity (showed by species richness, connectance, omnivory and mean FCL) and each environmental factor, which include ecosystem size (*S*), resource productivity (*R*) and disturbance extent (*D*) in 100 initial complex food webs (no loops, no cannibalism and the number of basal species $n_{P}\geq3$) generated by niche model. The competitive intransitivity of basal species $RI=1$ ($H_{ij}=0$ or 1). Other parameters see Fig. S30.
